# Walking Secure: Safe Routing Planning Algorithm and Pedestrian’s Crossing Intention Detector Based on Fuzzy Logic App

**DOI:** 10.3390/s21020529

**Published:** 2021-01-13

**Authors:** José Manuel Lozano Domínguez, Tomás de J. Mateo Sanguino

**Affiliations:** Department of Electronic Engineering, Computer Systems and Automatics, University of Huelva, Av. de las Artes s/n, 21007 Huelva, Spain; jose.lozano@diesia.uhu.es

**Keywords:** crossing intention detector, Android application, road safety, smart cities, safe routes, pedestrians

## Abstract

Improving road safety through artificial intelligence is now crucial to achieving more secure smart cities. With this objective, a mobile app based on the integration of the smartphone sensors and a fuzzy logic strategy to determine the pedestrian’s crossing intention around crosswalks is presented. The app developed also allows the calculation, tracing and guidance of safe routes thanks to an optimization algorithm that includes pedestrian areas on the paths generated over the whole city through a cloud database (i.e., zebra crossings, pedestrian streets and walkways). The experimentation carried out consisted in testing the fuzzy logic strategy with a total of 31 volunteers crossing and walking around a crosswalk. For that, the fuzzy logic approach was subjected to a total of 3120 samples generated by the volunteers. It has been proven that a smartphone can be successfully used as a crossing intention detector system with an accuracy of 98.63%, obtaining a true positive rate of 98.27% and a specificity of 99.39% according to a receiver operating characteristic analysis. Finally, a total of 30 routes were calculated by the proposed algorithm and compared with Google Maps considering the values of time, distance and safety along the routes. As a result, the routes generated by the proposed algorithm were safer than the routes obtained with Google Maps, achieving an increase in the use of safe pedestrian areas of at least 183%.

## 1. Introduction

Currently, smart cities are becoming a reality thanks to the use of information and communication technologies (ICTs), which allow to improve the services offered to their inhabitants [1]. Wireless communications together with data analysis and processing techniques such as big data, machine learning and artificial intelligence (AI) are promoting the development of technologies applied to smart cities, among other approaches [2,3,4].

An important area of smart cities is intelligent transport systems (ITS). These are made up of a set of technological solutions designed to coordinate, improve and increase transport safety on public roads [5,6]. At present, road safety of pedestrians represents a weak point of smart cities as stated in a study carried out by the General Administration of Traffic (DGT), in Spain. This study describes that there was a total of 13,545 accidents in urban areas involving pedestrians and vehicles in 2018, of which 5483 (40.48%) were pedestrians run over while crossing the street in the right place [7]. This number of outrages is not a unique concern of Spain. For example, a total of 71,000 run-overs occurred in the U.S. during 2017, of which 8.42% ended in death according to the annual report of the National Highway Traffic Safety Administration (NHTSA) [8]. These values are significant, not only because of the high number of accidents but also because of their upward trend as demonstrated by the Fatality and Injury Reporting System Tool (FIRST) [9].

As a result of this data, there is a growing concern about pedestrian road safety. In this field, there are several solutions in the state of the art based on the use of mobile applications aimed at improving road safety through different approaches. An example is the use of mobile devices to send information between vehicles and alert drivers on traffic jam situations in cities. For example, there is a solution that indicates the presence of road incidents using an algorithm that makes decisions based on a list of incidents. This method does not make use of context information, which could facilitate the calculation and detection of incidents [10]. A similar proposal to the previous ones is presented in [11]. It uses Google Maps and Google Directions to determine the best routes; it also uses contextual and historical information to estimate the probability that a route has incidents or not. Another example is the application developed to determine the risk of a driver, cyclist or pedestrian having an accident [12]. To this end, the app uses sensory fusion combined with a history of data and information entered by the users (e.g., age, weight and quantity of alcoholic beverages consumed). The use of apps on mobile devices also includes route customization within cities according to the specific needs of users (e.g., avoiding slopes up or down, reducing the distance traveled, etc.). In this way, this app avoids exposing the user to certain health risks. Besides, this app generates routes that require travelling a shorter distance than those offered by Google Maps thanks to the use of Open Street Maps (OSM) and the A * algorithm [13]. In addition to the previous application, there is another proposal called *UniBS4All* based on Google Directions, which allows generating routes adapted to the needs of people with physical disabilities. To do this, the points of the routes are modified to avoid architectural barriers stored in a database. Nevertheless, it does not remove all barriers to visually impaired people [14]. Another approach developed for people with visual difficulties is found in [15]. This app uses a Dijkstra algorithm to calculate the best possible route based on people’s preferences and limitations, as well as the traffic congestion and dynamic obstacles on the route. The main limitation of this application resides in its algorithm, since Dijkstra consumes a lot of resources to calculate a route. The route optimization has also been focused from a personal point of view, allowing routes to be customized considering the user preferences. To do this, the eligible preferences are the number of green areas, number of social places, noise of the streets and total length of the journey. Later, routes are calculated thanks to an algorithm based on weights and OMS [16]. This application does not consider the hours of the day when the query occurs, which could modify the routes to avoid dark places at night or very crowded places during the day.

Other studies focus on road safety around crosswalks, especially on pedestrian detection. An approach in line with this paper is the detection of the person’s intention to cross using a camera-based system and machine learning algorithms that monitor zebra crossings [17]. This study shows that the “you-only-look-once” (YOLO) scheme offers higher performance than the traditional histogram of oriented gradients (HOG) and Haarcascade schemes. Despite this, when pedestrians are partially hidden, there is a drop in performance. With the same approach, several systems capable of detecting pedestrians while crossing a zebra crossing have been proposed [18,19]. They combine cameras and machine learning techniques such as region-based convolutional neural networks (CNN), vector support machines (SVM) or multilayer perceptron neural networks (MLP). These studies show that machine learning based on support vector machines (SVM) and the use of cameras are adequate to detect the presence of pedestrians in zebra crossings. Another solution also supported by cameras consists in analyzing the body movements and orientation of the person’s head to determine whether a pedestrian intends to cross the public road or not [20]. The best performance was obtained by a combination of CNN and SVM. This suggests that the contextual information is very useful to determine the intention to cross of a pedestrian. In this sense, long short-term memory (LSTM) neural networks using images and characteristics (i.e., gender, walking direction and group behavior) has been proposed to estimate the crossing intention with great accuracy. Nonetheless, this results in a slightly high false positive rate when trying to classify the type of pedestrian movement [21]. Another approach is a crossing intention detector based on the use of cameras onboard vehicles, which can determine—in addition to the intention to cross—if a pedestrian is crossing or standing, as well as if he/she is turning or beginning to cross. This is achieved thanks to the use of random forest (RF) and SVM, resulting in faster detection than traditional methods [22]. Another study used laser imaging detection and ranging (LIDAR) sensors along with dense neural networks (DNNs), CNN or recurrent neural networks (RNNs) to detect the pedestrian’s crossing intention [23]. The best model is DNN, which offers a significant improvement over SVM. Another solution is based on the joint use of cameras and laser sensors. The implemented techniques are long short-term memory with an attention mechanism (AT-LSTM) and SVM, corresponding the best results to AT-LSTM even in small intervals of time [24]. A complete list of features on the several state-of-the-art approaches described above is given for comparison purposes (Table A1, Appendix A).

With the aim of contributing to improve road safety, this work presents an AI-based application developed on Android that detects the intention of pedestrians to cross zebra crossings and creates safe routes throughout the city. Among the advantages, the solution allows to detect the people’s crossing intention at all points of a city, unlike camera-based or LIDAR sensor systems that operate at fixed specific points of the road. Besides, the proposed solution offers robustness against adverse weather or low visibility conditions, as it only uses the internal sensors of a smartphone. Moreover, the proposed solution is based on fuzzy logic, which generates a low computational cost compared to those approaches described in the state of the art to detect the intention of a pedestrian to cross streets. In contrast, solutions based on cameras or LIDAR sensors can suffer under these conditions (e.g., snow or rain). Moreover, the deployment of the solution proposed has a zero cost for the municipalities or private entities that manage the cities because the system is implemented in the own citizens’ mobile devices.

To sum up, the novelties proposed in this manuscript regarding the state of the art are (i) the development of a fuzzy logic approach with low computational cost to detect the pedestrian’s crossing intention through the own smartphones’ built-in sensors; (ii) the development of an optimization algorithm for calculating, tracing and guiding people through safe routes within a city considering pedestrian areas such as crossings, streets and walkways; (iii) in case of detecting the pedestrian’s intention to cross, the app has the additional capability to communicate with a luminous intelligent crosswalk previously developed by this research team [25]. As a result, such an intelligent crosswalk creates a light barrier to alert drivers so they can safely stop their vehicles. According to the contributions, this manuscript has been structured as follows. Section 2 describes the fuzzy-based crossing intention detector and its implementation. Section 3 shows the experimentation performed and the main results obtained. Finally, Section 4 presents the conclusions and future works.

## 2. Mobile Application Description

**Figure 1:**
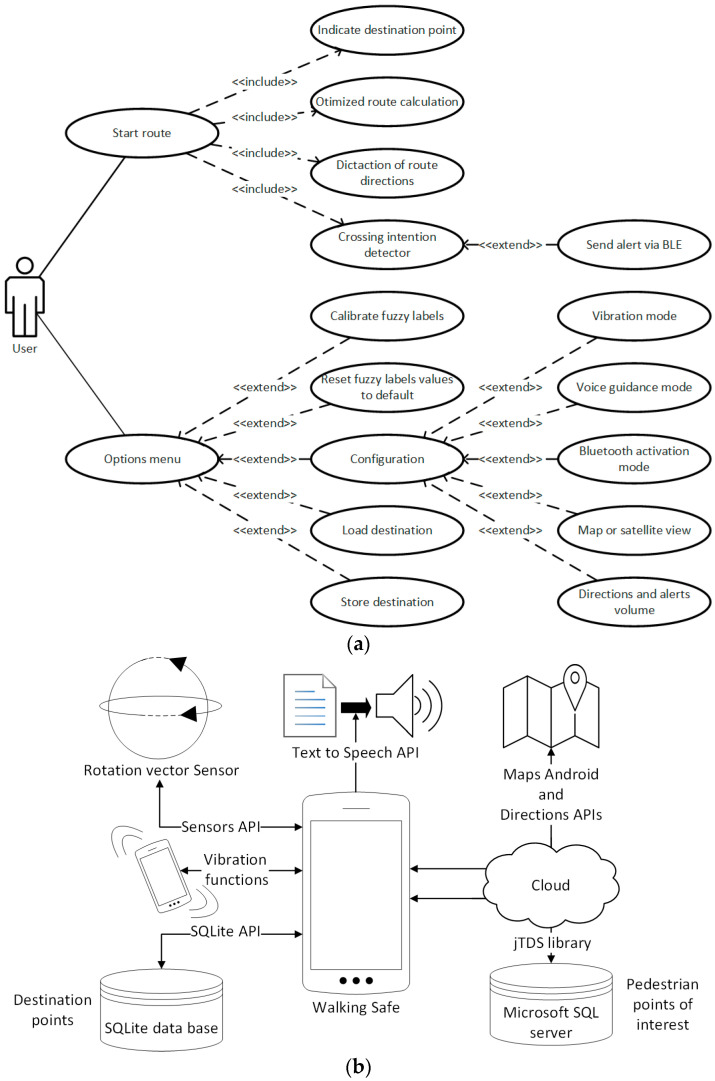
(**a**) Application functionalities. (**b**) Application architecture.

The mobile app has been developed through the Android Studio Integrated Development Environment (IDE). This programming environment allows developing smartphone applications in Java, C/C++ or Kotlin language. The app developed includes the following features: (i) calculation and tracing of safe routes for pedestrians through a city; (ii) safe guidance of people with hearing and visual impairments using haptic, visual and acoustic signals; (iii) detection of the pedestrian’s intention to cross zebra crossings; (iv) visualization aid system for pedestrians at zebra crossings through the use of Bluetooth communications. The app’s functionalities are summarized through the use case diagram shown in Figure 1a.

The app developed makes use of the application programming interfaces (APIs) of the Android operating system. At least, version 5 of the Android operating system is required for the execution of the app on a mobile device. This limitation is imposed by the APIs used in the app development, these being the following: Android maps, Directions, TextToSpeech and Sensors. In addition to these APIs, the application makes use of the SQLite API to manage a local database (i.e., destination points) and the jTDS library [26] to query an external database hosted on Microsoft SQL Server (i.e., pedestrian points of interest). The use of APIs requires the use of a network, and the app itself uses a database hosted in the cloud, so a constant connection to the Internet is needed. The app architecture and interaction with the APIs is shown in Figure 1b. 

The installation file of the mobile app takes 3345 KB of disk space, and requires 9.37 MB in the phone memory. It has been determined through the Android Profiler tool of Android Studio that the main thread of the app consumes 20.03% of the app’s runtime process, and 20.94% of RAM is used by the application. The app was monitored for 60 s, and it used the CPU for 25.95 s (44.46%) during this time. The highest CPU usage belongs to the graphical environment with 16.79% of the total CPU usage, followed by the main thread of the application with 8.91% of usage. During monitoring, it was determined that the total use of the RAM was 106.5 MB, of which 44.1 MB belongs to the graphical environment and 22.3 MB to the application code.

### 2.1. Calculation, Tracing and Guiding of Safe Routes

Among the main features of the application is the ability to calculate and plot safe maps in cities. The calculation and tracing of these maps are based on the Directions APIs and Android Maps, both from Google. The process for calculating a route begins with the detection of the user’s location through the Global Positioning System (GPS). Once the current position has been obtained, the user can indicate the destination to which he/she wants to go using two options. The first one is to select the destination location by clicking on the map and then activating the start of the route. The second option is obtained by pressing the route start button that will prompt the user to enter the address and city of the destination point by text. Once the origin and destination points of the route are known, an algorithm responsible for calculating, optimizing, and plotting safe routes is invoked (Algorithm 1); this process is done in the background to avoid congesting the main process thread. The optimization algorithm starts with getting the default route provided by the Directions API from the origin and destination points. Once the default route is known, a query is made to an external database located in the cloud, which stores pedestrian points of interest (e.g., zebra crossings, walkways and/or pedestrian areas). The jTDS library is used to carry out these queries. Subsequently, the algorithm calculates possible pedestrian points of interest near the default route that would increase the safety of the route. To do this, the points should not be more than 30 m away from the route in order to not to increase the distance excessively. Similarly, the total distance of the route to be traveled will never be more than 300 m from the original route. Once the points of interest have been determined in the first query, a second query is made to the Directions API to get a new route including the origin and destination points along with the points of interest obtained from the previous step. This route optimization is iteratively calculated to reach the safest possible route without an excessive increase of the distance to be traveled by the user.**Algorithm 1****Purpose:**obtaining a safe route to go from an origin point to a destination point**Inputs:**origin and destination points**Output:**representation of the route on a city map and guidance of the user’s safe route1: routePoints ← get the default route (origin, destination)2: pedestrianPoints ← pedestrian areas near to the route (routePoints)3: **if** size (pedestrianPoints) ≠ 04:optimizedRoute = false5:**repeat**6:routePoints ← route optimization (origin, destination, pedestrianPoints)7:pedestrianPoints ← calculation of new pedestrian points near to the route (routePoints)8:**if** size(pedestrianPoints) = 09: optimizedRoute = true10:**end if**11:**until (!optimizedRoute)**12:**end if**13: trace safe route on city map14: highlight the safe pedestrian points included in the route by means of a special icon

Once the final route is calculated, it is traced onto the map using Android Maps and the corresponding guidance instructions are stored. These instructions include both the waypoints of the default route (e.g., “go straight on”, “turn right”, “turn left”) and the specific points introduced by the application (e.g., “you are approaching a pedestrian zone”). These instructions are dictated by the app with verbal language as users move along the path generated, as well as indicated by haptic signals through the smartphone (i.e., vibrations). These methods have been implemented to enhance the user experience and facilitate notice to people with visual and/or hearing impairments, as well as to elderly people. To generate the indication correctly, the application waits until the user is near the waypoint while moving along the route. Once the user is in the zone, the directions are dictated using the TextToSpeech API. This API allows to convert text into a human voice, a function that has been specially designed for people with visual impairments. In addition to this option, guidance by vibration has also been implemented for people with hearing impairments to alert on areas where the route direction should be changed (e.g., taking a zebra crossing, walking down a street or pedestrian walkway). This functionality is based on the use of the basic vibration functions implemented on Android and can be configured in the options menu of the application to set how often the instructions should be dictated. The setting of each warning mode (i.e., voice-guided or vibration-driven) is independent, as is the volume at which the application transmits instructions.

### 2.2. Sensory Fusion

**Figure 2:**
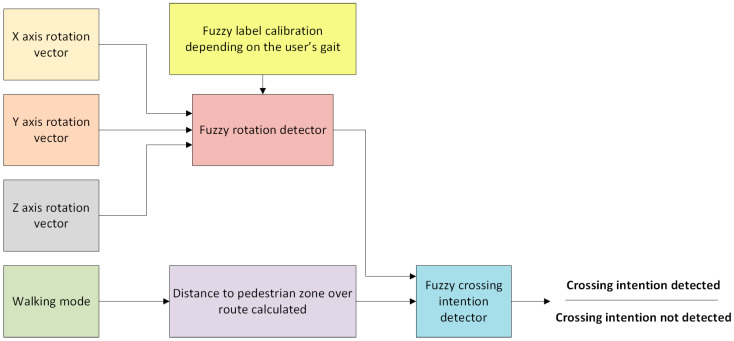
Sensory fusion scheme.

Unlike the different approaches described in the state of the art, the solution proposed herein determines the pedestrian’s crossing intention using sensory fusion. To do this, the flow diagram depicted in Figure 2 has been followed. The strategy addressed to detect the pedestrian’s crossing intention is based on: (i) the distance at which the user is located from a point of interest collected in the safe route (e.g., zebra crossing); (ii) walking mode established by the user in the app’s options menu; and (iii) a fuzzy rotation detector responsible for tracking the user movements. The main contribution of this work lies in the combination of these blocks to detect a pedestrian’s crossing intention around a point of interest. Each detail of the sensory fusion is explained in the following subsections.

#### 2.2.1. Fuzzy Rotation Detector

The block named “fuzzy rotation detector” has been constructed over the Android Sensors API. Specifically, this API asks the operating system for the sensor data related to the rotation vector; this can be hardware or software. The sensor is commonly used to measure movements and rotations, so it has been selected because the movements that pedestrians usually perform when approaching a crosswalk are rotational. In other words, pedestrians make turns as they approach a zebra crossing, being captured by this type of sensor.

To use it, it is necessary to enable the sensor in the main thread of the app, as well as to set the sample rate of the sensor. The frequency used for this application was 5 Hz (i.e., periods of 200 ms). To avoid saturating the main thread of the app due to the sample rate and the subsequent processing of the values, this task has been moved to an Android service. This represents a separate execution thread responsible only for processing the data generated by the sensor.

The data generated by the rotation sensor stands for the device’s angle variation—and therefore that of a pedestrian—at a specific time. To obtain a more accurate measurement of the pedestrian movement, the average value of a sliding window with 10 values was used. In this way, a total of 2 s of pedestrian movement is kept in memory. By averaging 2 s of movement, it is possible to determine if a pedestrian makes changes in his/her path or if he/she continues to walk straight.

The rotation is determined by using the “fuzzy rotation detector” block depicted in Figure 2, which considers the average variation of the rotation on the XYZ axes. The detector is based on fuzzy logic of Mamdani type with linguistic rules such as “If X1 is A1 and … X*n* is X*n*, then Y is B”, where the predictor and resulting variables have been established by an expert system [27]. The membership functions of the fuzzy set have been defined in a trapezoidal way—since its suitability to the data model is correct and not computationally complex—where the conjunction and implication operators utilize the minimum T-norm [28]. In addition, the First Infer, Then Add (FITA) method has been used for the defuzzification process because it is more consistent than the First Add, Then Infer (FATI) method [29]. Also, the voting method [30] has been used to produce a singleton (i.e., a single value), which results in a label that can be “go straight” or “rotation detected”. The set of fuzzy rules used for the rotation detector is included in Table 1. It can be seen in the rule set that, to determine that a user is rotating, at least a rotation on the *Z*-axis must occur. The user’s rotation is never determined if the *Z*-axis does not detect a rotation. This has been determined experimentally because, regardless of the position of the smartphone, the *Z*-axis always determines the rotation that the user performs on the ground. It is important to note that a rotation never exists on a single axis regardless of the other axes when a pedestrian walks.

**Figure 3:**
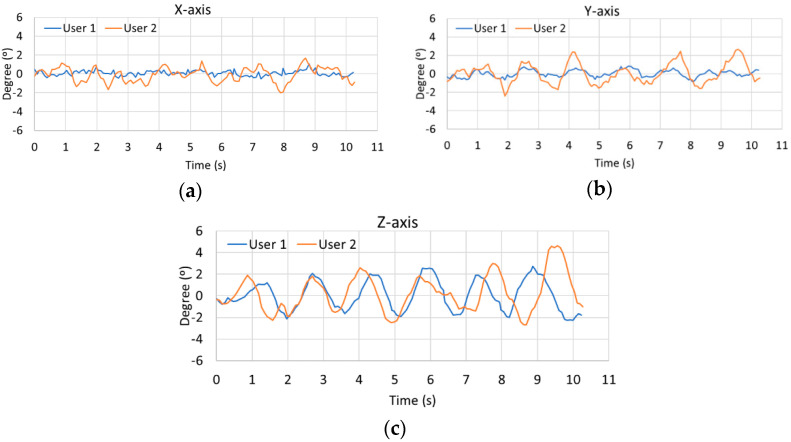
Graphical comparison of two pedestrian gaits over a straight line for 10 s. (**a**) *X*-axis comparison. (**b**) *Y*-axis comparison. (**c**) *Z*-axis comparison.

One advantage of the developed strategy is that the fuzzy labels used as inputs of the rotation detector are automatically established from the calibration of the sensors performed by the user. This process is necessary since, as shown in Figure 3, two different individuals have different gaits (i.e., walking styles) and generate different rotations. The graphs in Figure 3 show the rotation on the XYZ axes produced by two users as they take a straight line for 10 s. As seen in all cases, the first user produces more complex oscillations than the second user. As a result, it is found from this test that user 2 causes more obvious movements when walking than that generated by user 1. Complementarily, Table 2 lists the average, standard deviation, maximum and minimum values of each user for each axis of the graphs represented. These values comprehensively demonstrate that it is necessary to calibrate the fuzzy detector due to such high differences that exist between the two users when walking.

**Figure 4:**
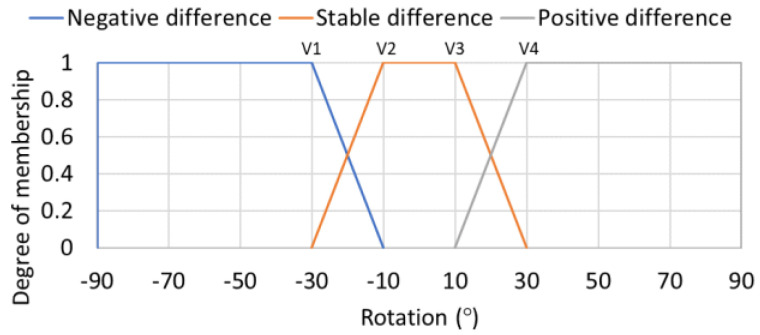
Example of fuzzy labels used for rotation detection inputs.

As a result of the previous comparison, the application has included a functionality that allows the automatic calibration of the smartphone sensors. The calibration task can be performed by the user through the options menu located in the upper left area of the app. Once the option is selected, the application indicates the user how to get a correct calibration. This consists in standing still for 5 s to avoid generating incorrect oscillations and then walking for 12 s in a straight line. At the end of the calibration, the maximum and minimum rotation values generated by a pedestrian for each of the XYZ axes are determined. From these values, the membership set of the input variables for each axis is determined as set out in Equation (1) and Table 3. The value “axisDifference” in Table 3 corresponds to the top plateau of the trapezoid in Figure 4 labeled as “Stable difference”. V1, V2, V3 and V4 values are calculated according to the form expressed in Table 3.

axisDifference = |minimum axis value| + |maximum axis value|(1)

#### 2.2.2. Fuzzy Crossing Intention Detector

**Figure 5:**
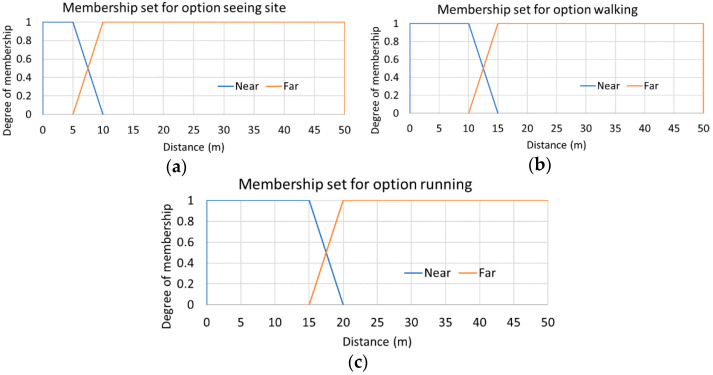
Fuzzy sets used to determine how far the pedestrian is from the next pedestrian point of interest base on its options. (**a**) Sightseeing; (**b**) walking; (**c**) running.

The output of the “fuzzy rotation detector” is used as the input for the block called “fuzzy crossing intention detector” that performs the system sensory fusion (Figure 2). Besides this, the sensory fusion requires—as input—the distance between the pedestrian areas and the optimal route calculated. The distance to the pedestrian zone with respect to the calculated route is computed from the current pedestrian position to the next pedestrian zone collected on the route. To this end, the user can select the walking mode from the following options: “running”, “walking” or “sightseeing”. This sets a greater or lesser distance to fix the time of detection around a zebra crossing (i.e., 15, 10 or 5 s). The membership functions used to calculate whether a pedestrian is near or far from the point of interest are shown in Figure 5, which are based on trapezoidal membership sets. The set of values of the membership functions is determined by selecting the walking mode that the user does. These fuzzy sets have been built considering a GPS sensor position error around one meter. In the worst case, the crossing intention can be safely determined with at least a distance of 5 m to the point of interest, although it could be determined from 7.5 m with enough certainty as shown in Figure 5a. Finally, as in the case of the rotation detector, the output is also a singleton that allows to indicate whether the intention to cross a crosswalk has been detected or not. The set of fuzzy rules used in this case is listed in Table 4. This sets that the pedestrian crossing intention will only be detected when he/she is near the next pedestrian point of interest on the safe route and he/she is also performing a rotation.

### 2.3. Pedestrian Visualization Aid System

**Figure 6:**
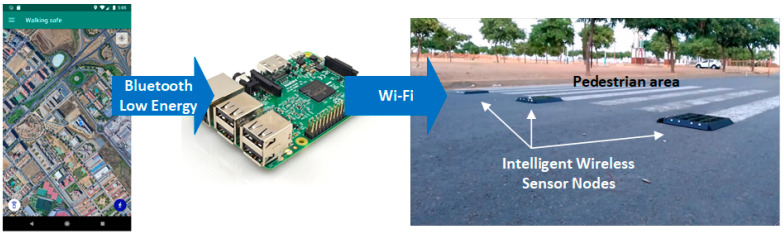
Communication scheme used between the application and the smart crosswalk.

Additional functionality has been included to add value to the app. This can communicate with a smart crosswalk as the one described in [25] to alert drivers about the presence of pedestrians with the intention to cross a zebra crossing. The interaction between the app and the intelligent crosswalk is managed by a gateway that can communicate with the app via Bluetooth and with the nodes of the intelligent crosswalk via Wi-Fi (Figure 6). To this end, a Raspberry Pi 3 device has been selected to implement the functions of that gateway.

The app makes use of the Bluetooth Low Energy (BLE) service implemented in Bluetooth 4.0. For this purpose, a BLE server has been created in the mobile application to provide information about the pedestrian’s crossing intention. The service can send the values “intent to cross detected” or “intent to cross not detected” from the fuzzy detector described above. To do this, the app makes use of the basic BLE profile called Alert Notification Profile, a communication profile that implements the New Alert feature. The goal is to allow any BLE client to read the current state of the pedestrian phones regarding their crossing intention or subscribe to the service to receive any change of state.

In the gateway, a script that allows converting the Raspberry Pi 3 into a BLE client of the app has been implemented in NodeJS. The client relies on the Noble library [31] to make use of the BLE services, and on the dgram library [32] to make use of the Wi-Fi communication with the intelligent crosswalk network. The client running on Raspberry Pi 3 connects to the smartphones once they enter the Bluetooth range and subscribes to the New Alert feature offered by the app. In case the app issues a notification, it is processed by the client; if it indicates that the person intends to cross the intelligent crosswalk, the client sends an activation message to the smart nodes of the crosswalk to generate a visual barrier on the roadway that allows vehicles to stop safely, further increasing the safety of the smartphone’s users.

It is important to note that the application allows to indicate optionally how often the Bluetooth communication is activated. The options allow to keep Bluetooth always on or to activate it automatically when the smartphone is 30 m away from a crosswalk. This way, it is possible to reduce the energy consumption of the resources.

## 3. Experimentation

This section describes the experimentation performed with the application and its main results, which consisted of: (i) evaluating the fuzzy rotation detector; (ii) evaluating the sensory fusion strategy developed for the crossing intention detector; and (iii) comparing the performance of the safe route planning algorithm against the same routes generated by default with Google Maps. To carry out the experimentation, a BQ Aquaris V smartphone with an eight-core Qualcomm Snapdragon 435 processor (1.4 GHz), Adreno 505 graphics processing unit, 4 GB RAM and 64 GB storage capacity over Android 8.1.0 was used. This smartphone was distributed to all volunteers to carry out all the tests listed below.

### 3.1. Description of the Volunteers Set

The tests corresponding to the evaluation of the fuzzy rotation detector and the sensory fusion developed for the crossing intention detector have been carried out with a total of 31 volunteers, of whom 51.61% were men and 48.39% were women. The average age of the subjects is 39.74 years with a standard deviation of 13.85 years. The set of users has an average height of 168.74 cm and a standard deviation of 11.47 cm. If the subjects are analyzed according to gender, the group of men has an average height of 176.47 cm and a standard deviation of 8.59 cm, while the group of women has an average height of 160.47 cm in height and a standard deviation of 7.79 cm. The average age of the male group is 37.19 years and a standard deviation of 12.74 years, while the average age of the female group is 42.47 years and a standard deviation of 14.90 years.

### 3.2. Evaluation of the Fuzzy Rotation Detector

**Figure 7:**
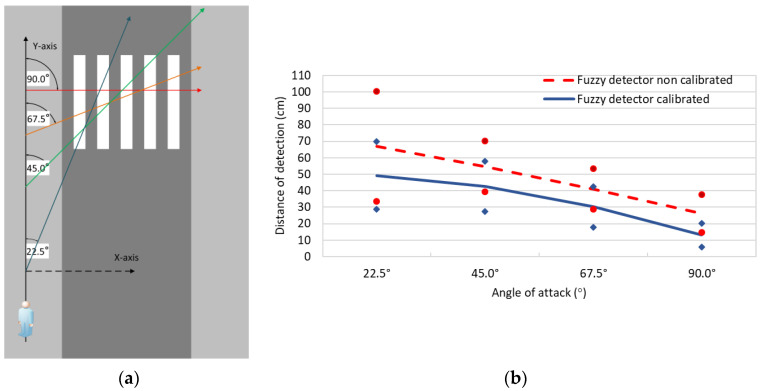
(**a**) Description of the test scenario. (**b**) Average results of the calibrated and uncalibrated rotation detector, where vertical lines represent the standard deviation for each average value.

To evaluate the fuzzy approach of the rotation detector, a series of tests have been designed to determine how far the pedestrian’s rotation is detected from the crosswalk considering a reference system (Figure 7a). The goal is to reproduce the conditions under which pedestrians typically cross a zebra crossing using different angles of attack. To do this, the rotations used have been 22.5°, 45°, 67.5° and 90°. As an example, the reference system used for the angle of attack of 22.5° has been represented in Figure 7a. Besides, the tests have been performed to compare the performance of the fuzzy detector both calibrated and uncalibrated.

The results show that the calibrated rotation detector offers a better response than the uncalibrated detector (Figure 7b). The main improvement is seen at 22.5°, where a reduction of 17.78 cm (26.51%) in the distance of detection of the calibrated detector versus the uncalibrated detector was obtained. Similarly, the calibration improved the detection distance in 12.04 cm (21.99%) for an angle of 45°, 10.90 cm (26.56%) for an angle of 67.5°, and 13.06 cm (49.84%) for a rotation of 90°. As a result, it can be asserted that the automatically calibration of the fuzzy labels established in the fuzzy detector improves the detection of angles of the app. In addition, it can be affirmed that the percentage of improvement is greater the less perceptible is the angle of attack used by the pedestrian to cross. However, the detection distance is generally better the greater the angle of rotation used by a pedestrian (i.e., less error). This suggests that abrupt angles of rotation (e.g., 90°) are easier to detect than more subtle angles of rotation (e.g., 22.5°).

### 3.3. Evaluation of the Sensory Fusion of the Crossing Intention Detector

The performance of the crossing intention detector has been evaluated using a receiver operating characteristic (ROC) analysis as described in [33]. The experimentation was carried out in a real environment under controlled conditions similar to that of Figure 7a to obtain sensitivity against specificity of the detector. The performance has been measured by means of a confusion matrix of 2 × 2 elements that relates positive (*p*) and negative (*n*) values (Table 5). To do this, the detection of the intention to cross of a pedestrian is considered positive while the nondetection of the turn intention is considered negative.

The experimentation carried out has obtained a total of eight confusion matrices, which provide the performance of the sensory fusion for each of the angles shown in Figure 7a, both calibrated and uncalibrated. To this end, 390 samples were used for each confusion matrix, totaling 3120 samples. The distribution of individuals who have undergone the tests is as described in Section 3.1. To carry out the collection of the samples, each subject was provided with a mobile device with the Android operating system, the application installed and containing the route with the test crosswalk. First, each volunteer performed the tests with the uncalibrated device; subsequently, the fuzzy labels of the fuzzy rotation detector were calibrated, and the same tests were repeated with the turn detector calibrated.

The series of tests consisted of (i) walking straight without intending to cross a pedestrian crossing; (ii) walking straight and then crossing the crosswalk according to the rotation angle set in each confusion matrix (i.e., 22.5°, 45°, 67.5° and 90°). It was established that, to consider a sample as true positive, the intention to cross had to be detected before entering the crosswalk; it was considered a false negative if the intention to cross was not detected or if the intention to cross was detected once a pedestrian entered the crosswalk; it was considered a false positive if a pedestrian was walking straight (i.e., no intention to cross the zebra crossing); and finally, it was considered a true negative when a pedestrian was walking straight and no intention to cross was detected.

After the experimentation, the average accuracy for the uncalibrated crossing intention detector was 84.74% with an F1-Score of 87.77%, whilst the calibrated crossing intention detector obtained an average accuracy of 98.63% with an F1-Score of 98.97%. As seen, the sensory fusion of the calibrated crossing intention detector offers better performance than the uncalibrated detector in all case studies and improves all the metrics in Table 6. Specifically, the average rate of true positives is 98.27% for the calibrated crossing intention detector. This is an excellent and fairly accurate value given a precision rate of 99.7%. Moreover, we found that the specificity is quite high reaching a level of 99.39%. Due to this, it can be affirmed that the sensory fusion of the calibrated crossing intention detector has high sensitivity and specificity, which makes the app very suitable for real use in cities.

It can be said—looking into the results obtained by the sensory fusion of the calibrated crossing intention detector—that the best results are found when detecting the crossing intention with 90° rotation. On the contrary, less relevant metrics are observed when the sensory fusion of the crossing intention detector is related to 22.5° rotation. This confirms, as in the case studied in Section 3.2, that the detection of rotation at smaller angles is more difficult to identify and because of this, the performance is reduced. Despite this, the sensory fusion of the calibrated crossing intention detector offers a better metric in the worst case than the best case for the uncalibrated detector. The best results obtained with the crossing intention detector are those achieved at angles of 90°, where it is observed that a value of 100% is always obtained in all the metrics except in the false positive rate (FPR), which is 0%, as expected. The following better metrics are obtained with angles around 67.5°. In this case, it is observed how the true positive rate (TPR) is reduced a little—although it is still very good like the rest of the rates—indicating that there have been some turns that have not been correctly detected by the calibrated crossing intention detector. The metrics corresponding to the rotations with an angle of 45° are found as the following better results for which a decrease in the TPR is observed. However, the biggest concern could be considered that the FPR is no longer 0%. Therefore, it suggests that the classifier detected the crossing intention at the wrong time. The same occurs for angles close to 22.5°; since the FPR rate increased to 1.68, the rest of the metrics are also reduced. This way, the calibrated crossing intention detector achieves the least robust set of metrics around an angle of 22.5° (TPR of 94.64%, accuracy (ACC) of 95.67% and F1-Score 96.86%). This is due to, in these cases, the rotation produced by the user is very slight and difficult to detect by the sensors currently used. In the same way, a limitation could be that all volunteers reside in the province of Huelva (Spain). This could have a bias due to the way they walk or cross the pedestrian crossing because they belong to the same specific geographical area. Another limitation can also be the calibration of the fuzzy labels at a specific moment. This calibration could become invalid if the user changes their gait for reasons such as it starts to rain, or he/she receives a phone notification (e.g., call, SMS or WhatsApp). It is important to highlight that the limitations on precision generated by a GPS sensor have been eliminated with the use of the fuzzy logic as mentioned in Section 2.2.2. For all the above, it can be confirmed that the fuzzy logic strategy using a mobile phone as a crossing intention detector on public roads offers good results, being demonstrated by the data generated by the experimentation carried out.

### 3.4. Comparison of Routes Generated

To evaluate the contribution made with the routing algorithm developed in the mobile application, a cloud-hosted database was created with a total of 79 pedestrian points of interest. These test points represent zebra crossings, walkways and pedestrian streets in different cities of Spain and Portugal (i.e., Huelva, Seville, Bollullos Par del Condado, Almonte, Camas, Tavira and Faro). In order to determine the safety improvement achieved by the routes traced with the application, these have been compared to the routes generated by Google Maps. The points of interest proposed have been used to demonstrate the errors made by Google Maps and how the proposed application improves the routes it generates.

The comparison makes it possible to study how Google Maps falls into various conflicting routes. For instance, it indicates pedestrians to circulate as if they were vehicles (e.g., using roundabouts), it does not direct people to take available pedestrian streets and it does not use walkways to cross three- or four-lane tracks. Table 7 shows a comparison of the routes generated by Google Maps versus the routes generated by the proposed algorithm. It shows the number of cases per route typology, average time difference, average distance difference, and average improvement achieved regarding safe zones. In summary, Table 7 shows that the routes calculated by the algorithm are safer than those generated by Google Maps by making use of a greater number of pedestrian zones. In contrast, the routes generated by the algorithm generally increase the time and distance of the path. The percent difference in each of these cases has been calculated as shown in Equation (2):

difference = (app route value ∗ 100)/(Google Maps route value) − 100(2)

In addition to these values, a composed metric has been proposed to evaluate the performance of the routes generated by the application against the routes generated by Google Maps. This metric has been called Composed Performance Metric (CPM) and it expresses the quality of the route—through a single value—as a function of the time, distance and number of safe pedestrian areas of the route. To make use of this metric, it is necessary for the values to be normalized from 0 to 1. For this, the min-max method described in [34] has been used. Once the values have been normalized, it is important to highlight that the values referring to the time and distance used in the route are better the closer to zero they are, while the values referring to the number of pedestrian areas are better the closer to one they are. Therefore, they will be represented by subtracting the normalized time and distance values from 1. Moreover, the number of pedestrian areas is represented according to the value obtained in the normalization. The metric is expressed mathematically by Equation (3):

CPM = (1 − normalized value of time) ∗ *K*_1_ + (1 − normalized value of distance) ∗ *K*_2_ + (normalized value of pedestrian areas) ∗ *K*_3_ (3)

where *K*_1_, *K*_2_ and *K*_3_ represent the weight for each of the values in the compound metric. The sum of *K*_1_, *K*_2_ and *K*_3_ must add up to 1. For the current case, an equitable weight has been used (i.e., *K*_1_, *K*_2_ and *K*_3_ have been set to 0.333). This compound metric supports modifying these weights to give greater importance to the factor to be highlighted (i.e., time, distance or safety). This metric has been used to evaluate each of the 30 routes studied, being better the closer its value is to 1. To this end, an average result for each type of route has been included in Table 7.

**Figure 8:**
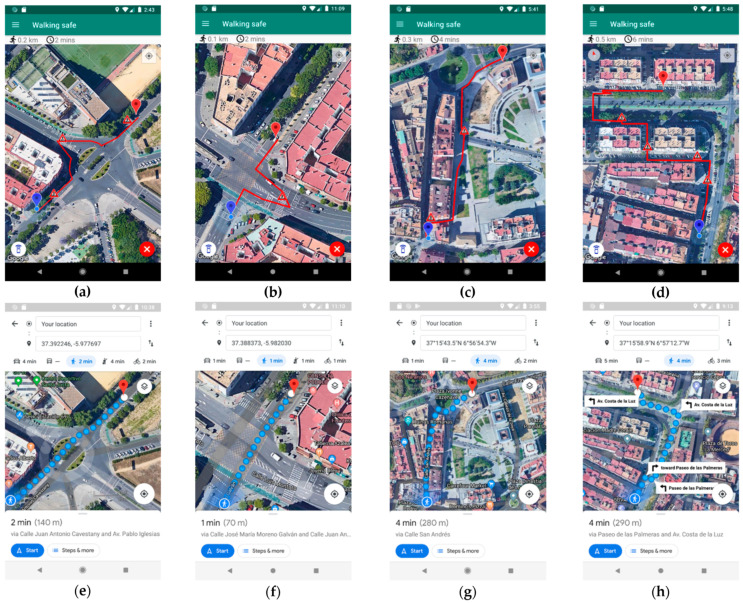
(**a**) Route calculated by the app where it is observed how to avoid the incorrect use of a roundabout. (**b**) Route calculated by the app where it is depicted how to avoid circulating like a vehicle. (**c**) Route calculated by the app where pedestrian areas are used. (**d**) Route calculated by the app where the pedestrian walkway is used. (**e**) Route calculated by Google Maps where a pedestrian is introduced into a roundabout. (**f**) Route calculated by Google Maps where a pedestrian is introduced into an intersection without respecting the signals. (**g**) Route calculated by Google Maps where pedestrians are not introduced through pedestrian areas. (**h**) Route calculated by Google Maps where the pedestrian walkway is not used.

Table 7 presents a comparison of the routes generated by the app developed and Google Maps. The first case shown in the table prevents a pedestrian from taking roundabouts as a vehicle, which is what happens with the routes generated by Google Maps. The routes generated by the app to avoid this scenario offer an improved safety of 243.75% compared to Google Maps, although this entails an increase in time and distance of 48.80% and 23.65%, respectively. In the same way, the app achieves a better CPM than Google Maps (0.65 and 0.62, respectively). The main improvement of this type of routes lies in indicating to pedestrians that they must move to the nearest zebra crossing instead of taking a roundabout as a vehicle (an example is shown in Figure 8a). On the contrary, Google Maps generates an unsafe route for the same origin and destination points. These route types are unsafe because pedestrians circulate through roundabouts regardless of whether they are walking in the opposite direction to the vehicles or if they are crossing the roundabout through the center, thereby generating a road safety problem for pedestrians and drivers, as it is shown in Figure 8e.

The second case aims to prevent a pedestrian from crossing public roads regardless of where the crossing is made. In other words, the application can generate routes that allow a pedestrian to avoid dangerous crossings by guiding pedestrians to zebra crossings so that they can safely cross the road. This type of route generated by the app obtains an average improvement in road safety of 183.00%, and in CPM (0.58 and 0.56, respectively). This also leads to an increase in time and distance required to complete the route (23.41% and 35.24%, respectively). An example of this route type is found in Seville (Spain), where, to cross a four-lane road, the developed app indicates the pedestrian to move to the closest crosswalk to avoid a possible run-over when crossing the street in the wrong place (Figure 8b). For this same crossing, Google Maps indicates the pedestrian to cross in a straight line regardless of his/her physical integrity. This poses a risk to the life and road safety of persons, which increases if they do not know the city, suffer from a visual problem, or have reduced mobility. The calculated route by Google Maps can be seen in Figure 8f.

The third case shows that Google Maps does not make efficient use of pedestrian streets in the cities, these being the ones that provide the most road safety to pedestrian because vehicles cannot circulate through them. In the proposed comparison, it is observed that the app manages to increase road safety by 215.48% on average compared to Google Maps, as well as reducing the distance to travel by 0.66%. Additionally, the CPM of the app is better than the CPM achieved with Google Maps (0.66 and 0.62, respectively). In this case, despite reducing the distance to travel, the Google Directions API does not show a reduction in time but an increase of 10.71%. An example of the aforementioned improvement is described in Figure 8c, where the app makes use of pedestrian areas such as the one that persons pass through. In this way, the road safety of pedestrians increases when walking through areas where there should not be cars circulating or parking. For the same route, Google Maps offers a trajectory where the use of pedestrian areas is not prioritized. In this case, Google Maps prefers to give the pedestrian a less safe route to travel a total of 20 m less than the route generated by the app, as it can be seen in Figure 8g.

Last but not least, the best results from the point of view of road safety comes from the routes that make use of pedestrian walkways to cross a road with three or four lanes. These routes improve safety by 266.67% on average. It is necessary to indicate that this increase in safety also increases the times and distance of the routes on average (147.62% and 129.26%, respectively). This is due to the need to move the user to the pedestrian walkways instead of crossing roads where the user’s integrity is in danger. These highly dangerous roads can be especially problematic for people with reduced mobility or with severe auditory or visual problems. In these cases, the route generated by the developed app provides even more significant safety than the route generated by Google Maps, despite the increased time and distance included. For these reasons, the CPM shows a tie between the routes generated by the app and Google Maps. An example of these routes is shown in Figure 8a, where it is observed how the pedestrian is indicated to move to the beginning of the pedestrian walkway used to cross a four-lane road that separates the origin and destination points. This way, the pedestrian needs to walk more time and a larger distance, improving at the same time its road safety. Moreover, when the pedestrian is in the most dangerous point of the route—near the access to a highway—he/she is at the safest point of the route since he/she is walking by a walkway only used for people. On the contrary, Google Maps provides a shorter distance and time, but it does not consider the road safety of the pedestrian. Thus, it tells the pedestrian to walk in the opposite direction of the vehicles on a road that does not have sidewalks. Google Maps also tells the pedestrian to walk along a road called “Av. Costa de la luz”, which is an access to join a highway. So, at the most dangerous point of the route, the pedestrian is more vulnerable to being run over because drivers do not expect to find a pedestrian on the access to a highway.

The limitations observed in this experimentation refer to the number of sites collected in an external database, since the ideal would be to have all the locations of pedestrian zones in the different cities studied and not just a sample of some dangerous locations. Another limitation refers to the drawing of the routes based on the Google Maps engine, which does not allow modifying the routes to make them pass on the sidewalk and always represents the routes on the road. Finally, based on the results obtained, it can be said that the routes generated by the app are safer than those generated by Google Maps. It has been achieved by guiding pedestrians for a longer time through reserved areas or by guiding them to a crossing where pedestrians have priority. Moreover, the total CPM shows that the routes generated by the app (0.64) are better in general than those obtained by Google Maps (0.61), since the increase in road safety provided by the application is greater than the increase in time and distance caused.

To finish, it is important to highlight that—to use the app—the smartphone always needs to be connected to the Internet because it uses several APIs and an external database that requires cloud connectivity. For this reason, we recorded the times taken by the app to calculate several routes based on the number of pedestrian points of interest and a 4G connection. The experimentation consisted in grouping the routes by the number of points of interest and averaging a series of 20 times. Accordingly, the results obtained were 1.9 ± 0.22 s to calculate the routes with one point of interest; 2.13 ± 0.37 s for routes with two points of interest; 2.13 ± 0.32 s for routes with three points of interest; 2.29 ± 0.28 s for routes with four points of interest; and 2.25 ± 0.41 s for routes with six points of interest. As a rule of thumb, the more points of interest added, the more time the algorithm takes to calculate the route. In this sense, the need for the Internet can be considered as a limitation in itself because, if the smartphone does not have connectivity to the cloud, the app cannot calculate the requested routes.

## 4. Conclusions

Smart cities are becoming a reality thanks to the support of wireless communications, along with the use of analysis techniques and data processing. Transport and road safety are an important pillar within smart cities. Currently, road safety is a weak point as demonstrated by several studies, which indicate that 40% of accidents involving pedestrians occur when people cross roads in the right place.

To help reduce accidents, this study presents a mobile application developed on Android that makes two contributions. On the one hand, the app can determine the intention of a pedestrian to cross through the public road using rotation sensors and sensory fusion based on fuzzy logic. This approach is integrated into the people’s smartphones. Therefore, the crossing intent detection system offers the advantage of being able to be used throughout the city instead of fixed specific points like other camera-based or LIDAR-based solutions. It should also be noted that the proposed solution—unlike other state-of-the-art systems—is robust against adverse weather conditions with low visibility, as it only makes use of simple sensors built into smartphones and requires no cameras. On the other hand, this work presented an algorithm for the calculation, tracing and guidance of pedestrians through safe routes. This includes the use of more zebra crossings, streets and pedestrian areas than other routing applications such as Google Maps. As an added value, the app has Bluetooth communication to interact with intelligent crosswalks and create a light warning barrier that allows drivers to safely stop their vehicles in case of detecting the crossing intention of pedestrians.

The experimentation carried out consisted in crossing a test zebra crossing with a set of 31 pedestrians using different entry angles, totaling of 3120 samples. From the tests, the fuzzy logic-based crossing intention detector has proven that it improves the crossing pedestrian intention detection after the automatically calibration of the fuzzy labels. Specifically, the accuracy rate of the calibrated fuzzy crossing intention detector was 98.63%, with an F1-Score of 98.97%, a true positive rate of 98.27%, a false positive rate of 0.61%, and a specificity of 99.7%. This suggests that the proposed solution has the capability to determine the intention of pedestrians to cross with high sensitivity and specificity.

Regarding the second contribution of this work, the experimentation carried out proved that the set of 30 routes computed by the proposed algorithm increased the safety of pedestrians against Google Maps between 183.00–266.67% by using a greater number of pedestrian areas (i.e., pedestrian crossings, streets and walkways). As a consideration, it should be mentioned that—in most cases—it entails an increase in the distance and time of the trips.

Future work focuses on improving the current functionalities of the app or include new ones. Among the possible improvements would be to implement an infrastructure-to-person (I2P) communication to alert on the presence of vehicles at high speed to avoid possible fatalities for pedestrians (i.e., communication with smart crosswalks). It also includes a person-to-vehicle (P2V) communication to notify the drivers’ smartphones about the existence of pedestrians intending to cross a crosswalk. In addition to this, another functionality to investigate is the possibility of changing the route calculation and map engines to OSM, which could offer more complete information about pedestrian areas in cities than Google Maps. Also, the possibility of using machine learning techniques to detect the crossing pedestrian intention with the mobile sensors will be studied, which could offer better performance than the current strategy. In this sense, one-class SVM techniques to detect anomalies, RNNs such as the long short-term memory (LSTM) to analyze time series or MLPs are candidates to be used as a crossing intention detector. In the second stage, the objective would be to increase the number of pedestrian interest points of the cities stored in the external database to increase the road safety provided by the app. In this task, the use of OSM can be very useful because it allows the community to update the maps of its city. In the third stage, the app would be uploaded to application stores such as Android’s Play Store or Apple’s App Store, among others. This would allow the massive download of the app by the users. In the fourth stage, the task would be to disseminate the app through traditional media (e.g., press or television) and social networks, as well as to attend entrepreneur fairs and exhibitions to publicize the product and its advantages for decision-makers. Finally, the results obtained from the research and the app itself would be transferred to third parties to continue with the maintenance of the project, either publicly or privately.

## Figures and Tables

**Table 1:** Rule set for the fuzzy rotation vector.

Rule Number	X-axis	Y-axis	Z-axis	Output
1	X-negative diff.	Y-negative diff.	Z-negative diff.	Rotation detected
2	X-negative diff.	Y-negative diff.	Z-stable diff.	Go straight
3	X-negative diff.	Y-negative diff.	Z-positive diff.	Rotation detected
4	X-negative diff.	Y-stable diff.	Z-negative diff.	Rotation detected
5	X-negative diff.	Y-stable diff.	Z-stable diff.	Go straight
6	X-negative diff.	Y-stable diff.	Z-positive diff.	Rotation detected
7	X-negative diff.	Y-positive diff.	Z-negative diff.	Rotation detected
8	X-negative diff.	Y-positive diff.	Z-stable diff.	Go straight
9	X-negative diff.	Y-positive diff.	Z-positive diff.	Rotation detected
10	X-stable diff.	Y-negative diff.	Z-negative diff.	Rotation detected
11	X-stable diff.	Y-negative diff.	Z-stable diff.	Go straight
12	X-stable diff.	Y-negative diff.	Z-positive diff.	Rotation detected
13	X-stable diff.	Y-stable diff.	Z-negative diff.	Rotation detected
14	X-stable diff.	Y-stable diff.	Z-stable diff.	Go straight
15	X-stable diff.	Y-stable diff.	Z-positive diff.	Rotation detected
16	X-stable diff.	Y-positive diff.	Z-negative diff.	Rotation detected
17	X-stable diff.	Y-positive diff.	Z-stable diff.	Go straight
18	X-stable diff.	Y-positive diff.	Z-positive diff.	Rotation detected
19	X-positive diff.	Y-negative diff.	Z-negative diff.	Rotation detected
20	X-positive diff.	Y-negative diff.	Z-stable diff.	Go straight
21	X-positive diff.	Y-negative diff.	Z-positive diff.	Rotation detected
22	X-positive diff.	Y-stable diff.	Z-negative diff.	Rotation detected
23	X-positive diff.	Y-stable diff.	Z-stable diff.	Go straight
24	X-positive diff.	Y-stable diff.	Z-positive diff.	Rotation detected
25	X-positive diff.	Y-positive diff.	Z-negative diff.	Rotation detected
26	X-positive diff.	Y-positive diff.	Z-stable diff.	Go straight
27	X-positive diff.	Y-positive diff.	Z-positive diff.	Rotation detected

**Table 2:** Comparison of the rotation values expressed in degrees (°) for each user while walking in a straight line for 10 s.

	User 1				User 2			
Axis	Avg.	Std. Dev.	Max.	Min.	Avg.	Std. Dev.	Max	Min
X-axis	0.079	0.278	1.080	−0.520	−0.089	0.772	1.670	−2.030
Y-axis	0.018	0.380	0.860	−0.830	0.046	1.010	2.680	−2.390
Z-axis	0.059	1.397	2.710	−2.280	0.222	1.702	4.630	−2.690

**Table 3:** Definition of the membership sets for each axis.

Label	V1	V2	V3	V4
Negative difference	−90°	−90°	Min. axis value − axisDifference	Min. axis value
Stable difference	Min. axis value − axisDifference	Min. axis value	Max. axis value	Max. axis value + axisDifference
Positive difference	Max. axis value	Max. axis value + axisDifference	90°	90°

**Table 4:** Definition of the membership sets for each axis.

Rule Number	Fuzzy Rotation Detector	Pedestrian Point of Interest	Crossing Intention (Output)
1	Go straight	Near	Not detected
2	Go straight	Far	Not detected
3	Rotation detected	Near	Detected
4	Rotation detected	Far	Not detected

**Table 5:** Confusion matrix for the receiver operating characteristic (ROC) analysis.

	Real Value		Total
	*P*	*N*	
Prediction			
*p′*	True positives (TP)	False positive (FP)	*P′*
*n′*	False negatives (FN)	True negatives (TN)	*N′*
Total	P	N	

**Table 6:** Results of the crossing intention fuzzy detector.

Fuzzy Crossing Intention Detector	Rotation Degree (°)	TPR (%)	FPR (%)	SPC (%)	ACC (%)	P (%)	F1-Score (%)
Non-calibrated	22.5	86.92	22.31	77.69	83.85	88.63	87.77
	45	98.83	18.05	81.95	71.28	91.37	94.95
	67.5	99.61	23.66	76.34	91.77	89.24	94.14
	90	100	23.85	76.15	92.05	89.35	94.37
	Average	96.34	21.97	78.03	84.74	89.64	92.81
	Std. Dev.	6.30	2.70	2.70	9.74	1.19	3.38
Calibrated	22.5	94.64	1.68	98.32	95.79	99.2	96.86
	45	98.85	0.77	99.23	98.97	99.61	99.23
	67.5	99.62	0.00	100	99.74	100.00	99.81
	90	100.00	0.00	100	100.00	100.00	100.00
	Average	98.27	0.61	99.39	98.63	99.7	98.97
	Std. Dev.	2.47	0.80	0.80	1.94	0.38	1.45

TPR: true positive rate; FPR: false positive rate; SPC: specificity; ACC: accuracy; P: precision.

**Table 7:** Comparison of routes generated by the algorithm proposed and Google Maps.

Typology of Route Tested	Cases Tested	Diff. in Time	Diff. in Distance	Increase in Safe Areas	App CPM	Google Maps CPM
Avoid roundabouts	8	48.80%	23.65%	243.75%	0.65	0.62
Avoid circulating as a vehicle	5	23.41%	35.24%	183.00%	0.58	0.56
Use pedestrian street	14	10.71%	−0.66%	215.48%	0.66	0.62
Use pedestrian walkways	3	147.62%	129.26%	266.67%	0.61	0.61
Total	30	36.68%	24.80%	222.72%	0.64	0.61

CPM: Composed performance metric.

## Data Availability

Not applicable.

## Appendix A

**Table A1:** Comparison of the proposals described in the state of the art.

Ref.	Type	Application	Information Extracted From	AI Implemented	**Year**
[10]	App	Traffic jams	Smartphone sensors	Decision algorithm in function of the via status	2018
[11]	App	Traffic jams and incidents	Smartphone sensors and third parties	-	2019
[12]	App	Accident risk	Personal information and historical data	Fuzzy harmonic systems and fuzzy patterns	2017
[13]	App	Improve pedestrian routes	Open Street Maps and Google APIs	A * algorithm	2018
[14]	App	Improve pedestrian routes	Data base with architectural barriers and Google APIs	Optimizing iterative algorithm	2020
[15]	App	Improve pedestrian routes	Maps stored in Google Drive and Sensors	Dijkstra algorithm	2020
[16]	App	Improve pedestrian routes	Open Street Maps	Optimizing algorithm	2018
[17]	Camera on the road	Detect crossing intention	Cameras	Haarcascade based on OpenCV library; HOG based on SVM; SSD based on MobileNet; YOLO based on DNN	2019
[18]	Camera on the road	Detect crossing intention	Cameras	Region-based CNN; SVM; MLP	2019
[19]	Camera on the road	Detect crossing intention	Cameras	HOG based on SVM	2017
[20]	Camera on the road	Detect crossing intention	Cameras	KNN; SVM; ANN; DT; CNN	2018
[21]	Camera on the road	Detect crossing intention	Cameras	LSTM	2020
[22]	Camera on board vehicles	Detect crossing intention	Cameras	RF; SVM	2017
[23]	LIDAR sensor on the road	Detect crossing intention	LIDAR	DNN; LSTM; CNN	2016
[24]	Cameras and laser sensor on the road	Detect crossing intention	Cameras and laser sensors	AT-LSTM; SVM	2020
Proposed	App	Detect crossing intention and improve pedestrian routes	Google APIs, external data base and rotation vector	Fuzzy logic and optimizing algorithm	2020

ANN: artificial neural network; AT-LSTM: long short-term memory network with attention mechanism; A* algorithm: A-Star search algorithm; CNN: convolutional neural network; DNN: dense neural network; DT: decision tree; HOG: histogram of oriented gradients; KNN: k-nearest neighbors; LSTM: long short-term memory; MLP: multilayer perceptron; RF: random forest; SSD: single shot detector; SVM: support vector machine; YOLO: you-only-look-once.
